# Intermolecular interaction as a direct measure of water solubility advantage of meloxicam cocrystalized with carboxylic acids

**DOI:** 10.1007/s00894-018-3649-0

**Published:** 2018-04-21

**Authors:** Piotr Cysewski

**Affiliations:** Department of Physical Chemistry, Pharmacy Faculty, Collegium Medicum of Bydgoszcz, Nicolaus Copernicus University in Toruń, Kurpińskiego 5, 85-096 Bydgoszcz, Poland

**Keywords:** Meloxicam, Cocrystals, Solubility advantage, Carboxylic acids, Intermolecular interactions, Aqueous solubility

## Abstract

The solubility advantage (SA) of meloxicam cocrystalized with mono- and dicarboxylic acids was expressed in terms of equilibrium constants involving active pharmaceutical ingredient and coformer in aqueous solutions. It is argued that SA can be quantified by concentration of pairs formed in water. The pH and concentration of dissolved components is included explicitly in the model. The alternative behavior of mono- and dicarboxylic acids was emphasized and addressed to different structural motifs. The structural and energetic properties of meloxicam and its complexes with carboxylic acids were characterized, including tautmerism and dissociation in aqueous media. In particular, performed in silico modeling confirmed experimental observation that meloxicam dissolved in water or modest acidic solutions is expected to be a mixture of anionic form in equilibrium with at least five neutral isomers. Tautomer-related diversity of pairs formation and the possibility of salt formation is also discussed.

**Electronic supplementary material:**

The online version of this article (10.1007/s00894-018-3649-0) contains supplementary material, which is available to authorized users.

**Graphical abstract:**
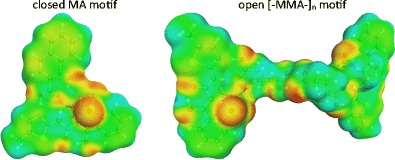
Two types of motifs found in meloxicam cocrystals reveal two sources of solubility advantage

## Introduction

Meloxicam is a modern drug commonly prescribed [1–3] for rheumatoid arthritis and osteoarthritis. It is also often used to mediate postoperative fever or pain and as an analgesic, especially where there is an inflammatory component. Meloxicam has a wider spectrum of anti-inflammatory activity, combined with less gastric and local tissue irritation than other nonsteroidal anti-inflammatory drugs available prior to its discovery [4]. In contrast with other medications of this type currently available, meloxicam appears to have greater inhibitory activity and selectivity against the inducible isomer of cyclooxygenase [5]. This is associated with a relief of gastrointestinal and renal events [3].

Unfortunately, meloxicam, which exists as a pastel yellow solid substance, is practically insoluble in water and because of this is classified as a II-class drug according to the Biopharmaceutics Classification System [6] suggesting that it has low solubility and high permeability [7]. Since the majority of orally administered drugs, including meloxicam, are taken as solid or semisolid dosage forms [8], the process of drug absorption must, in the first stage, involve dissolution of the released active pharmaceutical ingredient (API) in the gastrointestinal tract medium. Only after this step can permeation through biological membranes occur, driven by either a concentration gradient or an active carriage process [9]. Thus, the physiological process of transport into the bloodstream enabling further distribution, metabolism and excretion of any API is critically related to its solubility in bodily fluids. Due to very low solubility of meloxicam, the time to reach maximum concentration in the human body is about 4–6 h, taking more than 2 h for the drug to reach its therapeutic serum concentration [5]. This is one of the major shortcomings of meloxicam, reducing its potential of application for the relief of mild-to-moderate-level acute pain and long-term use to treat musculoskeletal disorders in humans and animals [10, 11].

The experimental values of meloxicam solubility in water differ significantly in different literature reports. Drug bank codes meloxicam as DB00814 and reports water solubility as low as 20.3 μM (7.15 mg dm^−3^) without providing the source and experimental conditions. In the Human Metabolome Database one can find record HMDB0014952, providing a much higher value for meloxicam aqueous solubility equal to 4.38 mM (154 mg dm^−3^). In a compilation by Yalkovith [12], two alternative measurements are reported at 25 °C and pH 6.0 suggesting values of water solubility of 65.0μM (22.8 mg dm^−3^) [13] and 34.2μM (12.0 mg dm^−3^) [14]. Such significant discrepancies between reported values of water solubility have become the norm, which is one of the major obstacles to formulating models for theoretical prediction of water solubility [15–17]. In the case of meloxicam, apart from the experimental protocol of solubility measurements, one of the reasons for such discrepancies is strong pH-dependent aqueous solubility, which can be related directly to its multiple ionization states [4, 18]. Meloxicam can be protonated at the thiazolic nitrogen atom and deprotonated at hydroxyl or secondary amine groups. The second source of divergence of solubility measurements can be ascribed to the potential variety of polymorphic forms of meloxicam [19]. Although only one solved structure of pure meloxicam has been deposited in the Cambridge Structural Database (CSD) [20] (SEDZOQ and SEDZOQ01) there are reports documenting the existence of up to five polymorphs. Unfortunately, the crystal structures of forms II and V claimed in US patent 6967248B231 [18] remain unknown so far, and the form denoted as IV is most probably not a polymorph but rather a monohydrate solid [19]. Interestingly, it seems that a contamination of form I with polymorphic form III, which apparently occurs spontaneously in formulated tablets of meloxicam, shows a higher solubility and greater intrinsic dissolution [19] than formulations containing only the dominant form I.

Low solubility always imposes problems from the perspective of bioavailability, reducing the therapeutic efficacy of any API. That is why this issue is one of the first improvements targeted in the development of new formulations of drugs. This can also be addressed to meloxicam. Several attempts to alter the pharmacokinetic profile of meloxicam have been undertaken, which resulted in various formulations including polymorph control [18, 19, 21–23], salts formation [24–26] encapsulation with cyclodextrins [13, 14, 25, 27], solvates preparation [28], utilization of solid dispersions [29, 30], nanosuspensions [31], nanoethosomes [32], nanocrystals [33] and, last but not least, cocrystallization [34–37]. This last method is particularly interesting due to the fact that it allows for engineering of more soluble structures by tuning and designing intermolecular interactions. Although various cocrystals of meloxicam with carboxylic acids have been synthesized, not all structures have been solved and only some systems have been characterized quantitatively as solubility enhancers [34, 36]. Nevertheless, the observed correlation [36] between in vitro and in vivo data documented that meloxicam cocrystals can possess a faster dissolution rate, exhibit increased oral absorption and an earlier onset of action.

Taking this information as a starting point, the aim of this project was to explore the idea of direct relationships between the observed solubility advantage of meloxicam cocrystals and the intermolecular interactions stabilizing the binary complexes formed in aqueous solutions. It is expected that, in the case of strong binding of the API with excipient, the recognition pattern will be related directly to the solubility of multicomponent solids, given that the building blocks formed by the supramolecular heterosynthon are present both in solution and in the solid state. To the author’s best knowledge this is the first attempt at theoretical justification of the solubility advantage of meloxicam cocrystals formed with carboxylic acids. The author believes that the proposed method could be extended to a broad class of systems and could be used to screen new solubility enhancers of other APIs.

## Methods

### Solubility advantage computation

The solubility advantage of meloxicam cocrystallization is defined here simply by the decadic logarithm of the ratio of molar solubility of cocrystal (S_CC_) with respect to meloxicam (S_API_):

1 $$ {SA}^{\mathrm{exp}}=\log \left(\frac{S_{CC}}{S_{API}}\right) $$

Suitable experimental values come from measurements published by Weyna et al. [36]. These data were confronted with predicted solubility of corresponding pairs in water solution via quantum chemistry thermodynamic computations [38, 39]. All important data used in this paper are collected in Table 1.

**Table 1:** Data used for predicting solubility advantage of meloxicam cocrystalized with carboxylic acids

Code^a^	Cocrystal former	S^a,b^	SA^a^	pKa_1_	pKa_2_	ΔG_r_^c,d^	pK_r_^c^	pMA^est^	logβ	C_o_^b^
M	Meloxicam	0.17	–	4.18	–	2.45	1.73	–	–	0.1^e^
1	1-Hydroxy-2-naphthoic acid	0.34	0.31	2.70	–	−6.43	−4.53	1.05	−5.58	0.5
2	Salicylic acid	0.26	0.18	2.97	–	−6.96	−4.91	−0.30	−4.61	0.5
3	Succinic acid	0.21	0.09	4.20	5.60	−6.13	−4.32	−5.30	−3.33	0.5
4	4-Hydroxybenzoic acid	0.16	−0.02	4.54	–	−6.34	−4.47	−1.42	−3.04	0.5
5	Glutaric acid	0.13	−0.10	4.30	5.40	−4.59	−3.23	−3.22	−3.25	0.5
6	Maleic acid	0.23	0.13	1.91	6.33	−9.72	−6.85	−8.25	−5.44	0.5
7	l-Malic acid	0.14	−0.08	3.46	5.10	−5.45	−3.84	−3.58	−4.10	0.5
8	Benzoic acid	0.15	−0.04	4.20	–	−6.42	−4.52	−1.14	−3.38	0.5
10	Hydrocinnamic acid	0.16	−0.03	4.57	–	−5.69	−4.01	−1.00	−3.01	0.5
11	Glycolic acid	0.16	−0.02	3.60	–	−7.84	−5.53	−1.55	−3.98	0.5
12	Fumaric Acid	0.18	0.02	3.30	4.44	−7.09	−5.00	−5.72	−4.27	0.5

^a^As reported in [36]

^b^In mg ml^−1^

^c^Computed in this paper

^d^Expressed in kcal mol^−1^

^e^Saturated solution in experimental conditions

In the case of monocarboxylic acids, their equilibrium in aqueous medium in the presence of meloxicam can be described simply by the following reaction, allowing for straightforward computation of the concentration of complex AM formed between reacting species:

2 $$ HA+ HM\leftrightarrows {H}_2 AM\kern1.5em ,\kern1.75em pAM={pK}_r+ pA+ pM $$

where p stands for negative decadic logarithm of reaction constant (*K*_r_) and monocarboxylic acid (A) or meloxicam (M) concentrations. Since the extent of non-dissociated forms of acids depends strongly on the acidity of the solution it is necessary to include both pH and values of dissociation constants into the equation, which can be done via the elementary formula:

3 $$ {\upalpha}_A=\frac{1}{1+{10}^{pK_A- pH}}\kern2em ,\kern1.5em {\upalpha}_M=\frac{1}{1+{10}^{pK_M- pH}} $$

Consequently the final concentration of meloxicam complexes in solution at any pH can be computed as follows:

4 $$ pAM={pK}_r+p\upbeta $$

where the last term is an actual pH-related correction defined simply by the following term

5 $$ p\upbeta =-\mathit{\log}\left({c}_A^o\bullet \left(1-{\alpha}_A\right)\bullet {c}_M^o\bullet \left(1-{\alpha}_M\right)\right) $$

Details of the derivation are provided in the Supplemental Material. It is very important to note the straightforward relationships of pAM with initial concentrations of both API ©_o(HM)_) and excipient ©_o(HA)_). Since not all the coformers used are very soluble in water, the experimental conditions of measurement [36] might lead in some cases to saturated solutions. The systems studied here were prepared [36] by placement of 50 mg of each component into 100 ml of water but, in the case of 1-hydroxy-2-naphthoic acid, this proportion far exceeds the aqueous solubility reported [36], which is as low as 0.1 mg ml^−1^. The activities of dicarboxylic acids acting as excipients can also be described in similar manner but it is necessary to include both stages of dissociation. The final formula allowing for computation of complex concentration is the same as (4) but definition of the pH-dependent share of non-dissociated form should be computed by the following formula:

6 $$ {\alpha}_A=\frac{1}{1+{10}^{pK_{A1}+{pK}_{A2}-2 pH}+{10}^{pK_{A1}- pH}} $$

The values of equilibrium constants of complexation reaction were computed with an aid of COSMOtherm17 using the BP_TZVPD_FINE_C30_1701.ctd parameter set [40] and taking advantage of the COSMO-RS (conductor like screening model for real solvents) approach [38, 39]. It is worth mentioning that one can, in principle, compute affinities between anionic forms of both carboxylic acids and dissociated meloxicam. This is of course indirectly related to estimation of values of dissociation constants. Unfortunately, computing of these values even for such simple cases as carboxylic acids is still not accurate enough and the error introduced might negatively interfere with computations of p*K*_r_. That is why experimental values of p*K*_a_ for all considered species were used instead of estimated ones.

### Structure optimization

**Fig. 1:**
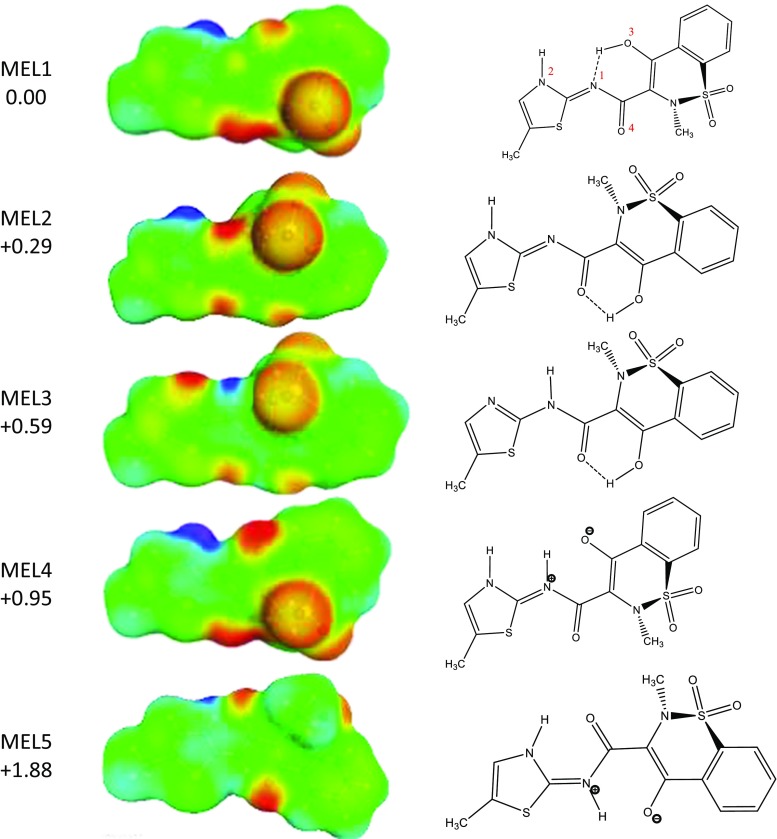
Structures and energetics of the most stable tautomeric forms of neutral meloxicam as found after modeling in water solution

Studying the thermodynamic properties of meloxicam in solution requires finding the most probable structures that fully characterize geometrical and energetic diversities. This step was achieved via extensive conformational analysis encompassing several conformational searches comprising possible tautomeric forms, such as for example enolic and zwiterionic forms. The geometries of all compounds were optimized both in the gas and condensed phases using BP-RI/TZVP scheme, which was followed by σ-profiles computation by means of the BP-RI/TZVPD approach in Turbomole v7.0 [41] interfaced with TmoleX 4.2 [42]. Since all monomers can potentially adopt many conformations, all important structures were included in the computations of thermodynamic quantities by explicitly considering those conformers/tautomers/zwitterions as generated using COSMOconf. The latter uses a multistage procedure generating hypothetical structures, the number of which was limited according to computed values of energy and similarity (RMSD). The default energetic window accepting conformers was raised to 20 kcal mol^−1^, and the number of generated structure was increased to 100. In practice these extensions were not necessary and only few low energy conformers were obtained for each compound. Initial structures of meloxicam complexed with carboxylic acids were taken from crystal structures if available. In the case of lack of suitable cif files, initial geometries of pairs were prepared manually by constructing of the recognition pattern comprising the carboxylic acid–azole supramolecular heterosynthon. Each complex was inspected for potential conformers and tautomers. The latter resulted from imposing alternative hydrogen bonding patterns via the –COOH group and re-optimization of geometries of the resulting pairs. Hence, conformational analysis also encompassed complexes of meloxicam with carboxylic acids. The inclusion of many potential structures resulted in covering of the wide range of conformation hyperspace lead to the set of the most probable structures both for monomers and binary complexes. The structures are provided below in Fig. 1 and Table 3.

## Results and discussion

The paper is organized by presenting the most important in-silico-derived properties of meloxicam followed by the characteristics of interactions with carboxylic acids in modeled water solutions. These data were then confronted with experimental solubility advantage. Hence, in the first part, structural and energetic analysis is provided for detailed characteristics of meloxicam properties in aqueous solution. Then, investigation of the diversity of hetero-synthon formed between API and excipients is provided. Finally, the titled problem is discussed, documenting observed relationships between stabilization of pairs in water solution with experimentally observed solubility advantage of meloxicam cocrystals.

### Tautomerism of meloxicam

Meloxicam shows polymorphism [4, 18, 19] and it is known that it can be isolated by crystallization from non-polar organic solvents such as tetrahydrofuran. This enolic form (often referred as form I of the solid state) is the one that is accepted as the most suitable for preparing pharmaceutical products [4]. However, another neutral structure of meloxicam in the form of zwiterionic tautomer has also been recognized. These structures were observed both in the solid state and in organic solvent solutions [4]. From a chemical point of view, the structure of meloxicam is very interesting due to its richness in potential tautomeric forms and structural flexibility, resulting in several conformers. This is also associated with the formation of two distinct types of inter- and intra-hydrogen bonding patterns related to its proton-donating and proton-accepting abilities. According to values of microacidities predicted by ChemAxon (MarvinSketch 16.9.12.0) the most acidic site corresponds to heterocyclic nitrogen atom N2 (see notation in Fig. 1) for which p*K*a = 0.5. The hydroxyl group has a typical phenolic character with an estimated p*K*a value is equal to 4.5. Finally, the nitrogen atom belonging to the acetamide bridge joining both aromatic fragments is the most basic site, with a p*K*a of about 10.6. However, computations of possible tautomeric structures in the COSMO model of water solution reveal interesting new features. The resulting lowest lying tautomers are presented in Fig. 1, and document the tautomeric richness of meloxicam. The most stable tautomer is found to be the enolic tautomer, which is stabilized by a hydrogen bonding motif of the N⋯H–O type. This, however, requires deprotonation of the N1 center and protonation of N2. It is worth noting that this form is not present in the crystal of pure meloxicam solids, but can be found in the crystalline monohydrates (CSD refcode WODBIA). Interestingly, this tautomer in monohydrate solid state is stabilized by a hydrogen bond formed between the water molecule and the N2 center. Unfortunately, due to the type of intramolecular hydrogen bonding, this tautomer is not ready for the heterosynthon formation with carboxylic acids via supramolecular pattern involving azole nitrogen centers.

**Fig. 2:**
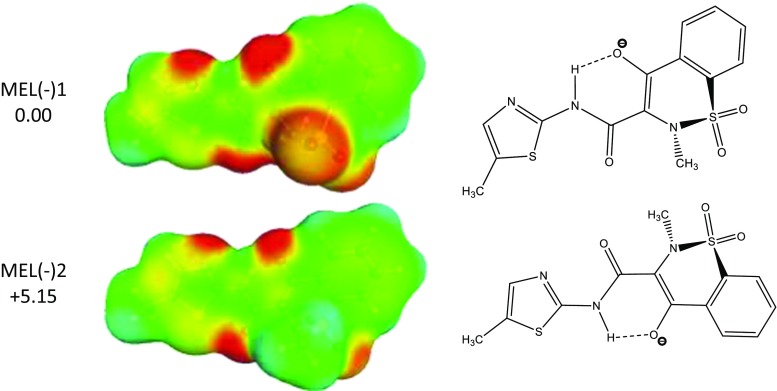
Structures and energetics of the most stable tautomeric forms of meloxicam anion as found after modeling in water solution

The second tautomer, which is higher in energy by only 0.29 kcal mol^−1^ is also protonated at the N2 site, but rotation of the main aromatic fragment along the acetamide chain allows formation of the alternative hydrogen bonding between two oxygen atoms. The tautomer MEL2 is ready for intermolecular complex formation with carboxylic acids, which actually will be observed in some pairs. In structures deposited under refcode SEDZOQ or SEDZOQ01, one can find a third tautomer denoted in Fig. 1 as MEL3, which favors meloxicam dimerization. In the case of such contacts, one of the oxygen atoms of the sulfonyl group interacts via hydrogen bonding with the N1 site of another meloxicam molecule and vice versa. On the other hand, the same tautomer is observed in the majority of meloxicam cocrystals since the intermolecular interactions favor direct formation of two hydrogen bonds of the –COOH group with thioazole N1 and secondary amine N2 nitrogen atoms. It is worth mentioning that this third tautomer is less favorable in water solution by only +0.59 kcal mol^−1^ with respect to MEL1. The fourth tautomer is in the form of the zwitterion obtained by proton transfer from O3 to N1, preserving protonation at the N2 center. This is associated with a rise in energy of +0.95 kcal mol^−1^ with respect to the most stable structure. Also the fifth tautomer included in Fig. 1 adopts a zwitterionic structure with an alternating intramolecular H-boding pattern induced by another conformation of the bridge connecting two vicinal heterocyclic rings, and is 1.88 kcal mol^−1^ above the most stable one. There are also other higher energy conformers of meloxicam that are not shown in Fig. 1. All this information suggests structural diversity of meloxicam and its complexity in water solutions, which is extended by including the ionic forms resulting from dissociation. The experimental value of pKa is equal to 4.18 [4], which univocally suggests that meloxicam is quite a strong acid and, in water solution, will exist predominantly in anionic forms. After deprotonation, several tautomeric forms can be considered, but apparently one predominates exclusively. As documented in Fig. 2, two anionic structures of lowest energy meloxicam are separated by more than 5 kcal mol^−1^, and they differ by the internal hydrogen bonding pattern. The anionic form is observed not only in aqueous solutions [4, 43] but is also detected in non-aqueous media in the presence of even traces of water [44, 45]. Finally, it is worth mentioning that, since the sulfuric group imposes non-planarity on the heterocyclic ring, there are two stereo-chemical configurations of the associated methyl-substituted heterocyclic nitrogen center. Since these structures are energetically equivalent they are not included in Fig. 1, but the pool of conformers considered in thermodynamic computations is doubled. Hence, meloxicam dissolved in water or modest acidic solutions is expected to be mixtures of practically one anionic form in equilibrium with at least five neutral isomers.

### Cocrystallization of meloxicam with carboxylic acids

Meloxicam can form multicomponent solids, and the majority of them comprise carboxylic acids. This encompasses cocrystallization of meloxicam with the following monocarboxylic acids: benzoic acid [36], 4-hydroxybenzoic acid [36], aspirin [34], 2-hydroxybenzoic acid [35, 36], 1-hydroxy-2 naphthoic acid [35, 36], hydrocinnamic acid [36], glycolic acid [36]; and the following dicarboxylic acids: acetylendicarboxylic acid [46], succinic acid [35, 37, 47], glutaric acid [34–36], l-malic acid [34–36], fumaric acid [36, 48], terephthalic acid [37, 47] and hexanedioic acid [35–37, 47]. The main structural motif observed in cocrystals is the supramolecular heterosynthon linking meloxicam moiety with –COOH group. There is, however, an important difference between recognition of mono- and dicarboxylic acids. Typically, the former contacts comprise a closed bimolecular building block while open chains are more characteristic of the latter. The types of intermolecular motifs found in solved structures of cocrystals are summarized in Table 2. The unimolecular stoichiometry is observed in all cocrystals of monocarboxylic acids, which comes from the pairs being stabilized by a strong bi-center recognition supramolecular heterosynthon. The ratio of dicarboxylic acid and meloxicam can also be 1:1 but cocrystals are often richer in acid molecules. There are also salts or mixed salt/cocrystal motifs. The collections presented in Fig. 1 and Table 2 suggest the necessity of considering three types of interactions in solutions of meloxicam with carboxylic groups by including tautmerism within heterosynthon and the possibility of salt formation. The results corresponding to the optimizations performed are collected in Table 3. The data provided suggest that, in the majority of cases, meloxicam interacting with carboxylic acids adopts a tautomeric form denoted as MEL3 in Fig. 1. Pairs comprising the MEL2 form are also possible, but they are usually slightly less stable. In two cases, namely for maleic and glycolic acids, salt formation is observed. The data presented documents how meloxicam tautmerism plays an important role also in the case of pairs formed with carboxylic acids.

**Table 2:**
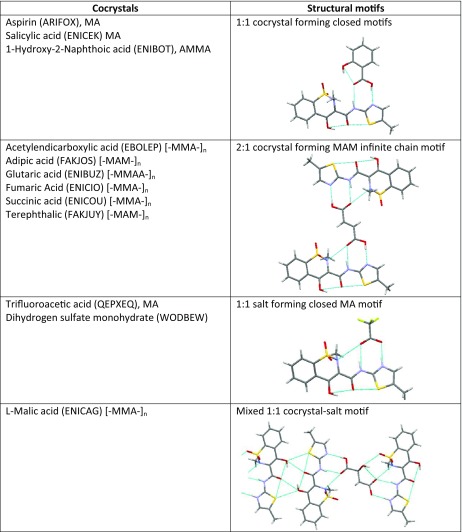
Collection of packing motifs stabilizing cocrystals formed by meloxicam (M) with carboxylic acids (A). The refcodes of solved structures deposited in the Cambridge Structural Database (CSD) are enclosed in brackets

**Table 3:**
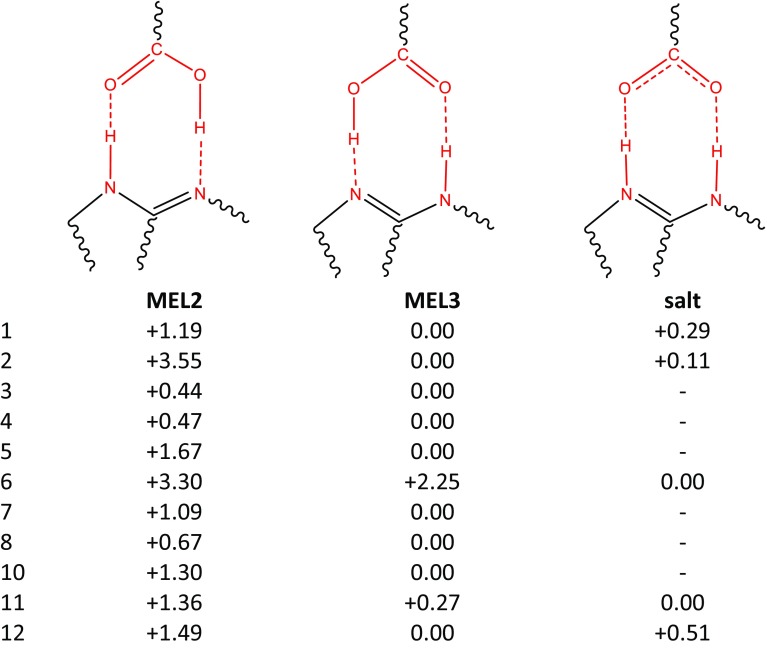
Schematic representation of heterosynthon structure with relative values of stabilization energy (in kcal mol^−1^) of pairs formed between meloxicam and studied carboxylic acids in modeled aqueous solutions

### Solubility advantage of meloxicam cocrystals

**Fig. 3:**
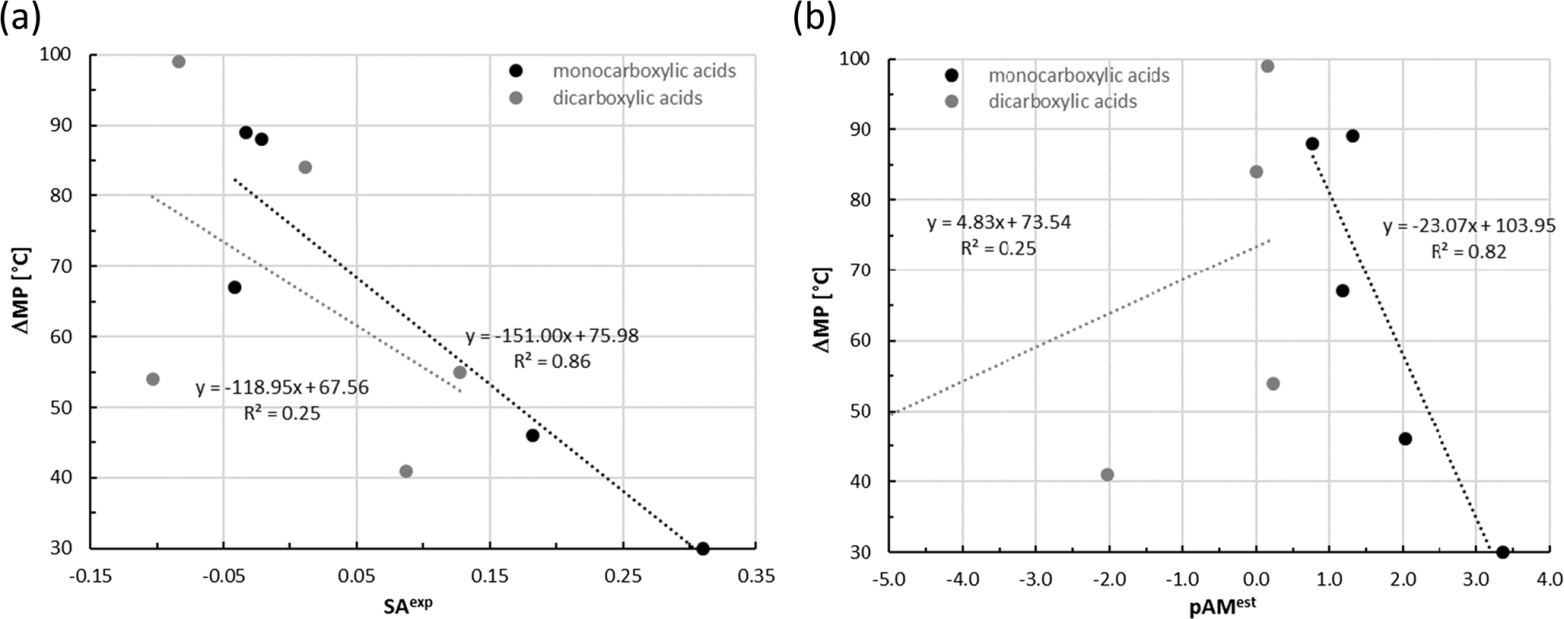
Diversity of trends between relative values of melting point and** a** experimental solubility advantage or** b** estimated values of decadic logarithm of concentration meloxicam complexes with carboxylic acids. The* ordinate* provides differences between melting temperature of cocrystals [35, 36] with respect of melting point of carboxylic acid

Firstly, it is interesting to note the existence of some experimentally observed trends between meting points (*T*_M_) and solubility advantage. In Fig. 3a the difference between cocrystals melting temperature with respect to the melting point of the coformer was plotted against experimental data [36] of the solubility advantage defined in Eq. (1). The positive values of SA obviously characterize systems enhancing meloxicam solubility and, conversely, negative ones denote the opposite consequence of cocrystallization. Figure 3a shows two distributions, presenting data separately for mono- and dicarboxylic acids. It is evident that two alternative rules governing dissolution of meloxicam cocrystals in water can be expected. The linear trend with at least qualitatively correlation (*R*^2^ = 0.86) is observed only for the former class of coformers, indicating that a systematic decrease of ΔMP^exp^ values is associated with the rise of experimentally observed solubility advantage of cocrystals. This stands for the very simple, fast and direct kind of zeroth approximation criterion needed for screening of coformers acting as potential solubility enhancers. Unfortunately, similar relationships were not detectable for a set of studied dicarboxylic acids, suggesting that the above rule is not general. The reason for this diversity might be due to the multitude of recognition patterns collected in Table 3. All monocarboxylic acids cocrystalize with meloxicam by forming pairs, which are building blocks that can be incorporated directly into the cocrystal. On the contrary, much bigger superstructures are typical for dicarboxylic acids interacting with meloxicam. This suggests that two mechanisms of solid formation for these two types of cocrystals are to be expected. Indeed, an attempt to correlate the estimated values of meloxicam concentrations (−pAM^est^) with experimental change of melting point observed upon cocrystallization also reveals two types of relationships, as documented in Fig. 3b. Again a linear relationship, of even better correlation (*R*^2^ = 0.91), exists only for monocarboxylic acids. It is necessary to mention that Fig. 3 shows collected values only for cases corresponding to an increase in cocrystals melting points with respect to coformers. Hence, two systems from the 12-case pool [36] were excluded from this analysis, namely 4-hydroxybenzoic and fumaric acids for which cocrystals melt at lower temperature than their coformers. Nevertheless, the plots presented in Fig. 3 document the different nature of cocrystallization of meloxicam with mono- and di-carboxylic acids. The relationships documented in Fig. 3 are quite encouraging and deserve further exploration.

**Fig. 4:**
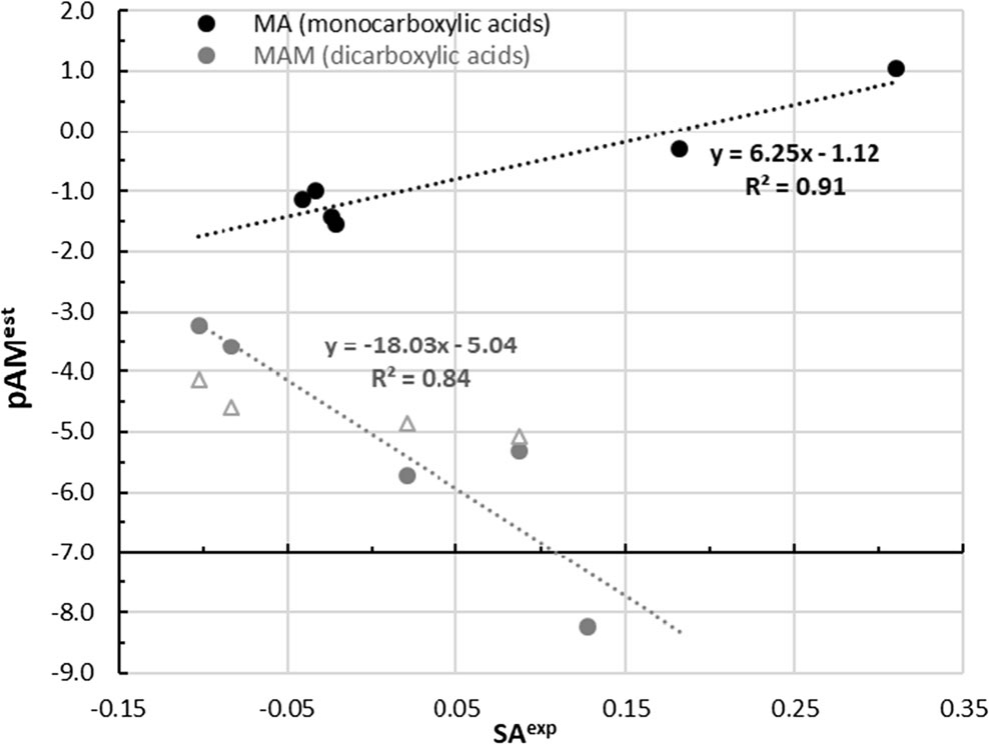
Correlation between estimated concentration of meloxicam complexes formed with carboxylic acids and the expediently observed solubility advantage of corresponding cocrystals

The experimental data against which computations are validated were measured at pH 6.5 and near normal body temperature, *t* = 37 °C. In such conditions, all carboxylic acids are almost completely dissociated. This is true for both mono- and dicarboxylic acids. Also, for meloxicam, the equilibrium is strongly shifted toward the anionic form. The pH-related correction collected in Table 1 (column logβ) quantitatively describes the cumulative effect of these two processes. This suggest that formation of the complex must overcome strong obstacles imposed by electrostatic interactions between both anionic forms of the interacting species. However, the affinity of meloxicam toward carboxylic acid and formation of the heterosynthon is so high that, even in situations of low concentrations of neutral forms, the existence of complexes is still probable. Indeed, the values of Gibbs free energies of reaction and associated equilibrium constants are quite high, as documented in Table 1. In all cases, the process is thought to be spontaneous even at acidic conditions. However, the estimated values of AM complex concentrations vary significantly for the carboxylic acids considered, which is seen clearly in the plots provided in Fig. 4.

The most intriguing is the observation of opposite trends for mono- and dicarboxylic acids. It is clear that the stronger solubility advantage of monocarboxylic acids is associated with higher values of pAM^est^. The opposite tendency is typical for dicarboxylic acids for which the rise of solubility advantage is paralleled with a decrease of estimated concentration of 1:1 complexes in aqueous solutions. Justification of these trends was already proposed by analysis of structural motifs characterizing two sets of considered cocrystals. Increasing the concentration of meloxicam complexed with monocarboxylic acid in aqueous solution contrasts with the rising driving force toward supramolecular structure formation based on the cocrystal building blocks appearing in solution. Although their concentration is relatively low, it is still higher compared to pure meloxicam, which explains the origin of solubility advantage. From these data, an experimental value of SA = 0.31 for 1-hydroxy-2-naphthoic acid can be derived. It is worth emphasizing that this acid is not characterized by the highest affinity toward meloxicam because there are two factors determining solubility advantage. Apart from the equilibrium constant, it is necessary to consider dissociation of all species under particular conditions in the solution, and 1-hydroxy-2-naphthoic acid is the strongest acid in the set analyzed. The situation involving dicarboxylic acids is slightly different. The higher solubility advantage is associated with an increase in concentration of 1:1 complexes. In these cases, crystallization requires the formation of much bigger superstructures for constituting the lattice. This very often must involve the attachment of a second meloxicam molecule. Table 4 provides structural and energetic details for selected cases, characterizing the smallest fragments beyond pairs.

**Table 4:**
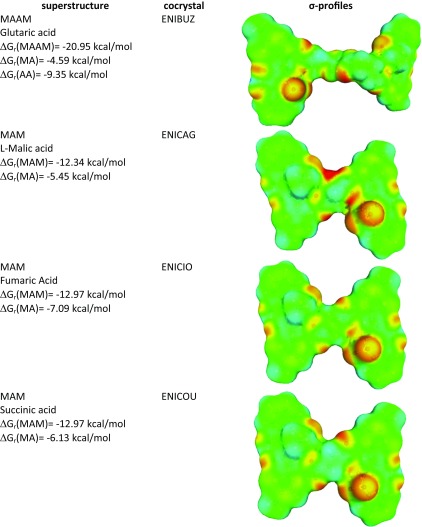
Supramolecular structures formed by selected dicarboxylic acids with meloxicam in aqueous solutions. The first value provided (Gibbs free energy of reaction) characterizes the affinity of the whole motive, while the remainder quantify the interactions of pairs

In the cases of l-malic, fumaric and succinic acids, such motifs can be coded schematically as [–MAM–]_n_. The inner part embraced by the bracket representing the repetitive fragment in the lattice is also thought to be stable in water solution. The Gibbs free energy of formation is fairly additive, as it is documented by the values provided in Fig. 4 and the position of the gray triangles in Fig. 4. Thus, it is not necessary to perform quite demanding computations of such big fragments in order to gain information about affinities; just pairs suffice for predictive purposes. Computations also confirmed mixed structure formation by l-malic acid bound to meloxicam. In this case, the complex found in water solution is the same as that observed in the solid state, and comprises one carboxylic group forming salt while proton transport toward meloxicam is not observed in the other group. In the case of glutaric acid, the smallest structural motif is bigger due to direct interactions of two carboxylic groups coming from different molecules. Hence, this case can be classified as [−MAAM–]_n_. Also for such a four-molecule system, the additivity of the values of Gibbs free energy is preserved fairly well.

It is also worth mentioning that the affinity of meloxicam toward dimer formation is quite disfavored in water solution. The Gibbs free energy is positive and corresponding equilibrium constant quite low. The estimated value of pM is as high as 4.8. This suggests not only that spontaneous formation of meloxicam dimers is quite unlikely but also that aggregations of bigger superstructures involving dicarboxylic acids and two or more meloxicam molecules is also thought to have low probability. This in turn makes it difficult to achieve the spontaneous formation of superstructures necessary for lattice formation, and multistep mechanism of cocrystallization is to be expected in the case of dicarboxylic acid cocrystallization. The low self-affinity of meloxicam has already been noted [47] and used as an argument for heterosynthon formation as a driving force toward meloxicam cocrystals formation.

### Relative values of hydration Gibbs free energy

Apart from the above relationships, it is worth mentioning that additional aspects might also affect the solubility advantage of meloxicam. One of the first properties that might be considered is the preferential solvation of a given species. For this purpose, the values of Gibbs free energies of solvation in water solutions were estimated for both pairs and monomers. Hence, as the measure of the relative hydration affinities, the differences between hydration Gibbs free energies of cocrystals and corresponding monomers were computed as follows:

7 $$ {\Delta  \Delta  G}_{CC}^{hydr}={\Delta  G}_{CC}^{hydr}-{\Delta  G}_M^{hydr}-{\Delta  G}_C^{hydr} $$

**Fig. 5:**
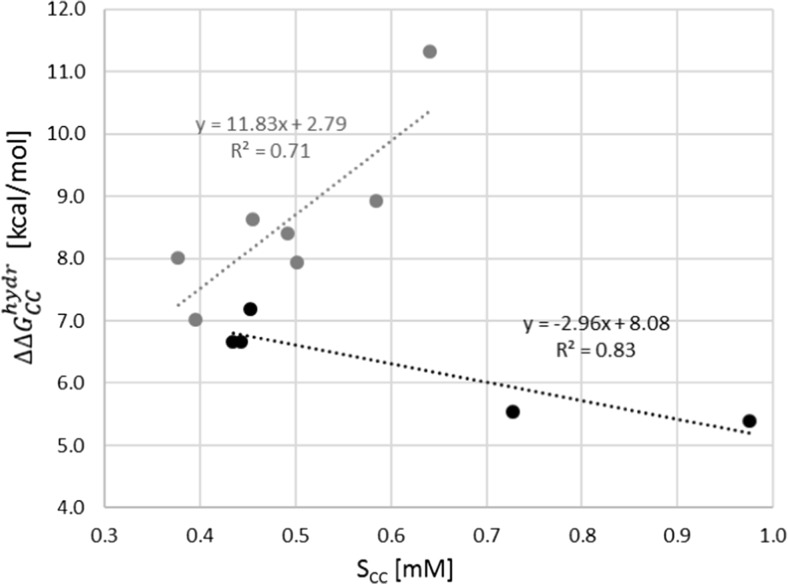
Relationships between relative hydration, ${\Delta  \Delta  G}_{CC}^{hydr}={\Delta  G}_{CC}^{hydr}-{\Delta  G}_M^{hydr}-{\Delta  G}_C^{hydr}$, of meloxicam cocrystals and solubilities

Positive values indicate higher affinity of monomers toward water solvent compared to pairs. As shown in Fig. 5, this is the case for all systems considered here, which can be attributed to the fact that the most polar fragments of interacting species are those involved in the intermolecular complex formation. Consequently, these molecular fragments of high affinity toward water become inaccessible for water molecules, which is directly responsible for their diminishing hydration energetics. Although solvation is strongly disturbed by formation of every pair, some interesting trends can be observed. In the case of pairs of meloxicam with dicarboxylic acids, one can notice a positive correlation of ${\Delta  \Delta  G}_{CC}^{hydr}$ with cocrystal solubility. Interestingly, the opposite tendency is associated with complex formation of meloxicam with monocarboxylic acids. Hence, the most soluble cocrystal is, at the same time, characterized by the lowest loss of Gibbs free energy of solvation. The correlation in this cases is as high as* R*^2^ = 0.83 and additionally explains the high solubility advantage gain by meloxicam by cocrystallization with 1-hydroxy-2-naphthoic acid. The trend characterizing bi-carboxylic acids has lower statistical significance. In this case, it is reasonable to expect that the loss of hydration is compensated for by the presence of a highly hydratable second carboxylic group.

## Conclusions

The problem of solubility advantage as one of the fundamental faced during the development of new forms of drugs was addressed here for the case of meloxicam cocrystalized with carboxylic acids. The main motivation of undertaking this research was the formulation of an in silico modeling protocol suitable for predicting of new systems that could offer gains in bioavailability inferred from solubility profiles. Despite the fact that available experimental data are limited, the in silico experiments performed can still offer valuable insights into cocrystallization mechanisms, allowing solubility advantage to be predicted, and meaning that interests are not restricted only to experimentally analyzed systems. Results of completed computations allowed quantification of meloxicam-carboxylic acid concentrations in aqueous media. The experimental conditions of solubility measures required detailed analysis of dissociation of both cocrystal formers and API. As a result, not only was a correlation between predicted values of complexes concentrations and observed solubility advantage provided, but the different behavior of mono- and dicarboxylic acids was also emphasized. Although the observed trends are semi-quantitative, they can still be used for screening of new coformers that enhance solubility of meloxicam in aqueous solutions. Dissolution, as a complex and multistage phenomenon, is governed by many factors and forces. However, as it was documented by performed computations this process can be partly rationalized in terms of equilibrium constants involving API and coformer in aqueous solutions. It was argued that the solubility advantage can be expressed as a function of pairs formation in water solution as one of the major contributions quantifying the dissolution process. Even if one achieves only modest correlations, this information is important since the affinity measure between API and coformer can be treated as a valuable molecular descriptor in QSPR studies. Meloxicam belongs to very broad class of drugs for which the proposed methodology of screening more soluble solid form can be applied directly. Furthermore, the heterosynthon comprising the two-point recognition pattern between carboxylic acid and the azole fragment occurs in other types of drugs. On the other hand, it is worth mentioning that, despite diverse values of equilibrium solubility advantage, all meloxicam cocrystals exhibited faster dissolution rates in the early phase followed by a decrease in meloxicam concentration over time. This property, termed the “spring-and-parachute” dissolution profile [49, 50], can also be addressed directly to formation of complexes in water solution. It seems reasonable to conclude that one of the stabilizing factors of the metastable state indispensable for the initial rise in API concentration can be related to direct interactions in aqueous solution and formation of complexes with coformers. All these arguments suggest that further exploration of the titled idea for practical purposes of screening of new drugs formulations is worth the effort.

## Electronic supplementary material

ESM 1 (DOCX 15.5 kb)

## References

[1] Thompson JP, Sharpe P, Kiani S, Owen-Smith O (2000). Effect of meloxicam on postoperative pain after abdominal hysterectomy. Br J Anaesth.

[2] Engelhardt G, Homma D, Schlegel K, Utzmann R, Schnitzler C (1995). Anti-inflammatory, analgesic, antipyretic and related properties of meloxicam, a new non-steroidal anti-inflammatory agent with favourable gastrointestinal tolerance. Inflamm Res.

[3] Roughan JV, Flecknell PA (2003). Evaluation of a short duration behaviour-based post-operative pain scoring system in rats. Eur J Pain.

[4] Luger P, Daneck K, Engel W, Trummlitz G, Wagner K (1996). Structure and physicochemical properties of meloxicam, a new NSAID. Eur J Pharm Sci.

[5] Davies DNM, Skjodt NM (2012). Clinical pharmacokinetics of meloxicam. Clin Pharmacokinet.

[6] Takagi T, Ramachandran C, Bermejo M, Yamashita S, Yu LX, Amidon GL (2006). A provisional biopharmaceutical classification of the top 200 oral drug products in the United States, Great Britain, Spain, and Japan. Mol Pharm.

[7] Amidon GL, Lennernäs H, Shah VP, Crison JR (1995). A theoretical basis for a biopharmaceutic drug classification: the correlation of in vitro drug product dissolution and in vivo bioavailability. Pharm Res.

[8] Allen LV Jr, Popovich NG, Ansel HC (2014) Ansel’s pharmaceutical dosage forms and drug delivery systems, 9th edn. Lippincott Williams and Wilkins, Baltimore. 10.1017/CBO9781107415324.004

[9] Tozer TN, Rowland M (2006) Introduction to pharmacokinetics and pharmacodynamics: the quantitative basis of drug therapy. Lippincott Williams and Wilkins, Baltimore.

[10] Meloxicam for oral administration (1999) https://www.google.sr/patents/US6869948?dq=meloxicam&hl=nl&sa=X&ved=0ahUKEwiX9fWQ8OzWAhXBAxoKHeD3DEkQ6AEIPzAD (accessed October 24, 2017)

[11] Highly Concentrated Stable Meloxicam Solutions for Needleless Injection (2013) https://www.google.sr/patents/US20140113893?dq=meloxicam&hl=nl&sa=X&ved=0ahUKEwiX9fWQ8OzWAhXBAxoKHeD3DEkQ6AEIJDAA (accessed October 24, 2017)

[12] Yalkowsky SH, He Y, Jain P (2010) Handbook of aqueous solubility data. CRC, Boca Raton

[13] Charumanee S, Titwan A, Sirithunyalug J, Weiss-Greiler P, Wolschann P, Viemstein H (2006). Thermodynamics of the encapsulation by cyclodextrins. J Chem Technol Biotechnol.

[14] Sanemasa I, Wu JS, Toda K (1997). Solubility product and solubility of cyclodextrin inclusion complex precipitates in an aqueous medium. Bull Chem Soc Jpn.

[15] Hopfinger AJ, Esposito EX, Llinàs A, Glen RC, Goodman JM (2009). Findings of the challenge to predict aqueous solubility. J Chem Inf Model.

[16] Abramov YA (2015). Major source of error in QSPR prediction of intrinsic thermodynamic solubility of drugs: solid vs nonsolid state contributions?. Mol Pharm.

[17] Palmer DS, Mitchell JBO (2014). Is experimental data quality the limiting factor in predicting the aqueous solubility of druglike molecules?. Mol Pharm.

[18] Coppi L, Sanmarti M, Clavo M (2003) Coppi, crystalline forms of meloxicam and processes for their preparation and interconversion. https://www.google.com/patents/US6967248 (accessed October 23, 2017)

[19] Jacon Freitas JT, Santos Viana OMM, Bonfilio R, Doriguetto AC, de Araújo MB (2017). Analysis of polymorphic contamination in meloxicam raw materials and its effects on the physicochemical quality of drug product. Eur J Pharm Sci.

[20] Allen FH (2002). The Cambridge structural database: a quarter of a million crystal structures and rising. Acta Crystallogr Sect B Struct Sci.

[21] Hanna M, Shan N, Cheney ML, Weyna DR (2010) In vivo studies of crystalline forms of meloxicam. European Patnet application EP2244712A1. http://www.google.com/patents/EP2244712A1?cl=en

[22] Baboota S, Dhaliwal M, Kohli K (2005). Physicochemical characterization, in vitro dissolution behavior, and pharmacodynamic studies of rofecoxib-cyclodextrin inclusion compounds. Preparation and properties of rofecoxib hydroxypropyl β-cyclodextrin inclusion complex: a technical note. AAPS PharmSciTech.

[23] Naidu NB, Chowdary KPR, Murthy KVR, Satyanarayana V, Hayman AR, Becket G (2004). Physicochemical characterization and dissolution properties of meloxicam-cyclodextrin binary systems. J Pharm Biomed Anal.

[24] Fuhrman LC (2006) Ansel’s pharmaceutical dosage forms and drug delivery systems, 8th edn. Am J Pharm Educ 70:71 http://www.ncbi.nlm.nih.gov/pmc/articles/PMC1636965/

[25] El-Mahrouk G, Aboul-Einien MH, Elkasabgy NA (2009) Formulation and evaluation of meloxicam orally dispersible capsules. Asian J Pharm Sci 4:8–22

[26] Ki H-M, Choi H-K (2007). The effect of meloxicam/ethanolamine salt formation on percutaneous absorption of meloxicam. Arch Pharm Res.

[27] Ghorab MM, Abdel-Salam HM, El-Sayad MA, Mekhel MM (2004). Tablet formulation containing meloxicam and beta-cyclodextrin: mechanical characterization and bioavailability evaluation. AAPS PharmSciTech.

[28] Seedher N, Bhatia S (2003). Solubility enhancement of cox-2 inhibitors using various solvent systems. AAPS PharmSciTech.

[29] Chiou AHJ, Yeh MK, Chen CY, Wang DP (2007). Micronization of meloxicam using a supercritical fluids process. J Supercrit Fluids.

[30] Saleem MA, Bala S (2010) Formulation and evaluation of meloxicam solid dispersion incorporated topical gels. Int J Pharm Biol Sci 1(3):1–9

[31] Ambrus R, Kocbek P, Kristl J, Šibanc R, Rajkó R, Szabó-Révész P (2009). Investigation of preparation parameters to improve the dissolution of poorly water-soluble meloxicam. Int J Pharm.

[32] Ahad A, Raish M, Al-Mohizea AM, Al-Jenoobi FI, Alam MA (2014). Enhanced anti-inflammatory activity of carbopol loaded meloxicam nanoethosomes gel. Int J Biol Macromol.

[33] Ochi M, Kawachi T, Toita E, Hashimoto I, Yuminoki K, Onoue S (2014). Development of nanocrystal formulation of meloxicam with improved dissolution and pharmacokinetic behaviors. Int J Pharm.

[34] Cheney ML, Weyna DR, Shan N, Hanna M, Wojtas L, Zaworotko MJ (2011). Coformer selection in pharmaceutical cocrystal development: a case study of a meloxicam aspirin cocrystal that exhibits enhanced solubility and pharmacokinetics. J Pharm Sci.

[35] Cheney ML, Weyna DR, Shan N, Hanna M, Wojtas L, Zaworotko MJ (2010). Supramolecular architectures of meloxicam carboxylic acid cocrystals, a crystal engineering case study. Cryst Growth Des.

[36] Weyna DR, Cheney ML, Shan N, Hanna M, Zaworotko MJ, Sava V (2012). Improving solubility and pharmacokinetics of meloxicam via multiple-component crystal formation. Mol Pharm.

[37] Myz SA, Shakhtshneider TP, Tumanov NA, Boldyreva EV (2012). Preparation and studies of the co-crystals of meloxicam with carboxylic acids. Russ Chem Bull.

[38] Klamt A (2011). The COSMO and COSMO-RS solvation models. Wiley Interdiscip Rev Comput Mol Sci.

[39] Klamt A, Schüürmann G (1993). COSMO: a new approach to dielectric screening in solvents with explicit expressions for the screening energy and its gradient. J Chem Soc Perkin Trans.

[40] COSMOlogic (2016) COSMOthermX Version C30_1601, http://www.cosmologic.de

[41] TURBOMOLE V7.0 (2015) a development of University of Karlsruhe and Forschungszentrum Karlsruhe GmbH, 1989–2007, TURBOMOLE GmbH, since 2007; available from http://www.turbomole.com

[42] Steffen C, Thomas K, Huniar U, Hellweg A, Rubner O, Schroer A (2010) TmoleX-A graphical user interface for TURBOMOLE. J Comput Chem 31(16):2967–2970. 10.1002/jcc.21576 10.1002/jcc.21576 20928852

[43] Banerjee R, Sarkar M (2002). Spectroscopic studies of microenvironment dictated structural forms of piroxicam and meloxicam. J Lumin.

[44] Delgado DR, Holguín AR, Almanza OA, Martínez F, Marcus Y (2011). Solubility and preferential solvation of meloxicam in ethanol+water mixtures. Fluid Phase Equilib.

[45] Delgado DR, Jouyban A, Martinez F (2014). Solubility and preferential solvation of meloxicam in methanol + water mixtures at 298.15 K. J Mol Liq.

[46] Tantardini C, Arkhipov SG, Cherkashina KA, Kil’met’ev AS, Boldyreva EV (2016). IUCr, crystal structure of a 2:1 co-crystal of meloxicam with acetylendicarboxylic acid. Acta Crystallogr Sect E Crystallogr Commun.

[47] Tumanov NA, Myz SA, Shakhtshneider TP, Boldyreva EV (2012). Are meloxicam dimers really the structure-forming units in the “meloxicam–carboxylic acid” co-crystals family? Relation between crystal structures and dissolution behaviour. CrystEngComm.

[48] Aitipamula S, Banerjee R, Bansal AK, Biradha K, Cheney ML, Choudhury AR (2012). Polymorphs, salts, and cocrystals: what’s in a name?. Cryst Growth Des.

[49] Tiekink ERT, Vittal JJ, Zaworotko M (2010) Organic crystal engineering: frontiers in crystal engineering. Wiley, New York

[50] Bavishi DD, Borkhataria CH (2016). Spring and parachute: how cocrystals enhance solubility. Prog Cryst Growth Charact Mater.

