# Supplementary material for: Intermolecular interaction as a direct measure of water solubility advantage of meloxicam cocrystalized with carboxylic acids
Source: J Mol Model. 2018 Apr 21;24(5):112. doi: 10.1007/s00894-018-3649-0 (PMC5911280; doi:10.1007/s00894-018-3649-0)
Supplement: Supplementary file 1 — (DOCX 15.5 kb) [file 894_2018_3649_MOESM1_ESM.docx]

Supporting materials

**Intermolecular interaction as a direct measure of water solubility advantage of meloxicam cocrystalized with carboxylic acids**

Piotr Cysewski

Chair and Department of Physical Chemistry, Pharmacy Faculty, Collegium Medicum of Bydgoszcz, Nicolaus Copernicus University in Toruń, Kurpińskiego 5, 85-096 Bydgoszcz, Poland, piotr.cysewski@cm.umk.pl

**Derivation of equation used in predicting of solubility advantage of meloxicam cocrystallziaed with mono and dicarboxylic acids.**

*Dissociation of mono carboxylci acid*

$$HA⇆H^{+}+A^{-}$$

$$K_{A}=\frac{\left[ H^{+} \right]\cdot\left[ A^{-} \right]}{\left[ HA \right]}, \alpha_{A}=\frac{\left[ A^{-} \right]}{c_{A}^{o}},c_{A}^{o}=\left[ A^{-} \right]+\left[ HA \right]$$

$$\alpha_{A}=\frac{\left[ A^{-} \right]}{\left[ A^{-} \right]+\left[ HA \right]}=\frac{1}{1+\frac{\left[ HA \right]}{\left[ A^{-} \right]}}=\frac{1}{1+\frac{\left[ H^{+} \right]}{K_{A}}}=\frac{1}{1+{10}^{{pK}_{A}-pH}}$$

$$pA=-log\left( \left[ HA \right] \right)=-log\left( c_{A}^{o}\cdot(1-\alpha_{A}) \right)$$

*Dissociation of dicarboxylci acid*

$$H_{2}A⇆H^{+}+{HA}^{-}$$

$$HA^{-}⇆H^{+}+A^{-2}$$

$$K_{A1}=\frac{\left[ H^{+} \right]\cdot\left[ HA^{-} \right]}{\left[ H_{2}A \right]}$$

$$K_{A2}=\frac{\left[ H^{+} \right]\cdot\left[ A^{-2} \right]}{\left[ HA^{-} \right]}$$

$$K_{A1}\cdot K_{A2}=\frac{\left[ H^{+} \right]^{2}\cdot\left[ A^{-2} \right]}{\left[ H_{2}A \right]}$$

$$\frac{\left[ A^{-2} \right]}{\left[ H_{2}A \right]}=\frac{K_{A1}\cdot K_{A2}}{\left[ H^{+} \right]^{2}}$$

$$\alpha_{A}=\frac{\left[ A^{-2} \right]}{c_{A}^{o}},c_{A}^{o}=\left[ A^{-2} \right]+\left[ {HA}^{-} \right]+[H_{2}A]$$

$$\alpha_{A}=\frac{\left[ A^{-2} \right]}{\left[ A^{-2} \right]+\left[ HA^{-} \right]+\left[ H_{2}A \right]}=\frac{1}{1+\frac{\left[ HA^{-} \right]}{\left[ A^{-2} \right]}+\frac{\left[ H_{2}A \right]}{\left[ A^{-2} \right]}}=\frac{1}{1+\frac{\left[ H^{+} \right]^{2}}{K_{A1}\cdot K_{A2}}+\frac{\left[ H^{+} \right]}{K_{A1}}}$$

$$\alpha_{A}=\frac{1}{1+{10}^{{pK}_{A1}+{pK}_{A2}-2pH}+{10}^{{pK}_{A1}-pH}}$$

*Dissociation of meloxicam*

Since meloxicam can formally by treated similar to HA the relations are simply repetition of equations defining mono carboxylic acid dissociation.

$$HM⇆H^{+}+M^{-}$$

$$\alpha_{M}=\frac{\left[ M^{-} \right]}{\left[ M^{-} \right]+\left[ HM \right]}=\frac{1}{1+\frac{\left[ HM \right]}{\left[ M^{-} \right]}}=\frac{1}{1+{10}^{{pK}_{M}-pH}}$$

$$pM=-log\left( \left[ HM \right] \right)=-log\left( c_{M}^{o}\cdot(1-\alpha_{M}) \right)$$

*Complexation reaction*

$$HA+HM⇆H_{2}AM$$

$$K_{r}=\frac{\left[ H_{2}AM \right]}{\left[ HA \right]\cdot\left[ AM \right]}, pAM={pK}_{r}+pA+pM , pAM=-log\left( \left[ H_{2}AM \right] \right)$$

$$pAM={pK}_{r}-log\left( c_{A}^{o}\cdot\left( 1-\alpha_{A} \right) \right)-log\left( c_{M}^{o}\cdot\left( 1-\alpha_{M} \right) \right)={pK}_{r}-p$$

where

$$p=-log\left( c_{A}^{o}\cdot\left( 1-\alpha_{A} \right)\cdot c_{M}^{o}\cdot\left( 1-\alpha_{M} \right) \right)$$
